# (*E*)-9-(But-2-en-1-yl)-6-chloro-9*H*-purine

**DOI:** 10.1107/S1600536813010416

**Published:** 2013-05-11

**Authors:** Fredrik Lundvall, Jindrich Kania, Lise-Lotte Gundersen

**Affiliations:** a Centre for Materials Science and Nanotechnology, Department of Chemistry, University of Oslo, PO Box 1126, 0315 Oslo, Norway; b Department of Chemistry, University of Oslo, PO Box 1033, 0315 Oslo, Norway

## Abstract

The asymmetric unit of the title compound, C_9_H_9_ClN_4_, contains two mol­ecules. In the crystal, the mol­ecules are ordered in a chain-like fashion along the *a* axis, and form layers offset relative to the *C* plane by approximately 30°. This ordering does not, however, appear to be directed by classical hydrogen bonding.The allylic side chains of both independent mol­ecules are disordered, with occupancies of 0.870 (4) and 0.934 (3) for the major components. The disorder components represent two possible spatial orientations of the atoms around the C=C double bond.

## Related literature

For synthetic background and applications, see Kania & Gundersen (2013 ▶).
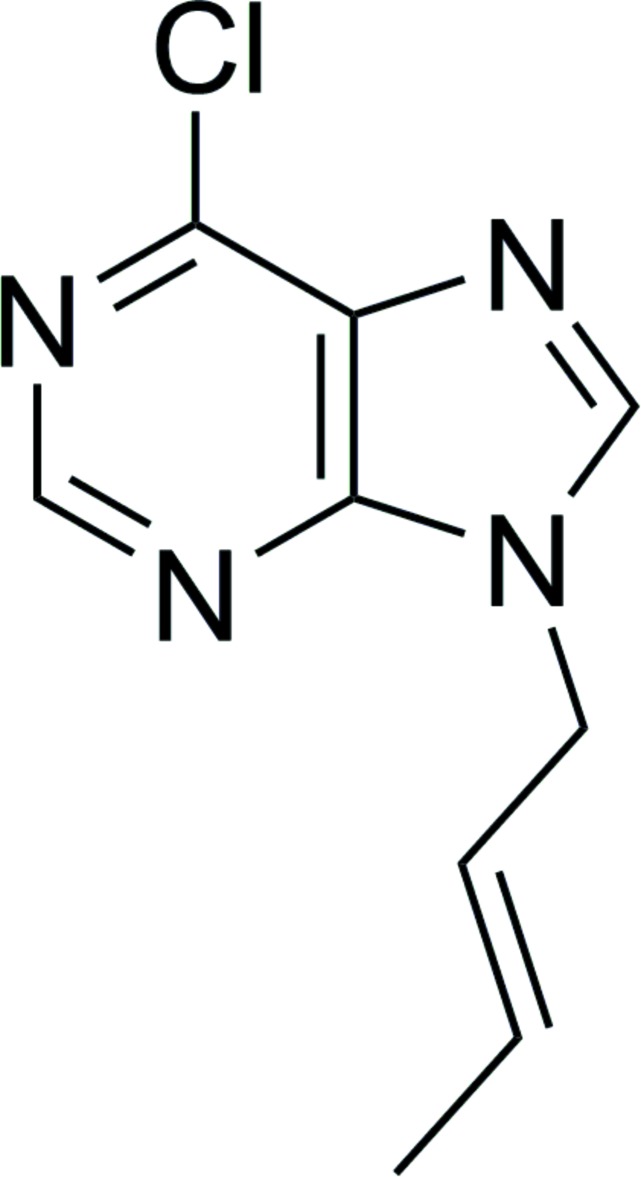


## Experimental

### 

#### Crystal data

C_9_H_9_ClN_4_
*M* *_r_* = 208.65
Triclinic, 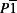

*a* = 8.1818 (11) Å
*b* = 9.7103 (13) Å
*c* = 13.9435 (19) Å
α = 69.642 (1)°
β = 75.448 (1)°
γ = 67.032 (1)°
*V* = 947.6 (2) Å^3^
*Z* = 4
Mo *K*α radiation
μ = 0.37 mm^−1^
*T* = 100 K
1.13 × 0.35 × 0.22 mm

#### Data collection

Bruker APEXII CCD diffractometer
Absorption correction: numerical (*SADABS*; Bruker, 2005 ▶) *T*_min_ = 0.683, *T*_max_ = 0.924
10801 measured reflections
4428 independent reflections
4103 reflections with *I* > 2σ(*I*)
*R*_int_ = 0.017

#### Refinement

*R*[*F* ^2^ > 2σ(*F* ^2^)] = 0.027
*wR*(*F* ^2^) = 0.070
*S* = 1.04
4428 reflections
283 parameters
4 restraints
H-atom parameters constrained
Δρ_max_ = 0.34 e Å^−3^
Δρ_min_ = −0.30 e Å^−3^

### 

Data collection: *APEX2* (Bruker, 2005 ▶); cell refinement: *SAINT* (Bruker, 2005 ▶); data reduction: *SAINT*; program(s) used to solve structure: *SIR92* (Altomare *et al.*, 1993 ▶); program(s) used to refine structure: *SHELXL97* (Sheldrick, 2008 ▶); molecular graphics: *DIAMOND* (Brandenburg, 2004 ▶) and *ChemBioDraw Ultra* (CambridgeSoft, 2009) ▶; software used to prepare material for publication: *publCIF* (Westrip, 2010 ▶).

## Supplementary Material

Crystal structure: contains datablock(s) I, global. DOI: 10.1107/S1600536813010416/lr2100sup1.cif

Structure factors: contains datablock(s) I. DOI: 10.1107/S1600536813010416/lr2100Isup2.hkl

Supplementary material file. DOI: 10.1107/S1600536813010416/lr2100Isup3.cml

Additional supplementary materials:  crystallographic information; 3D view; checkCIF report

## Figures and Tables

**Fig. 1.**
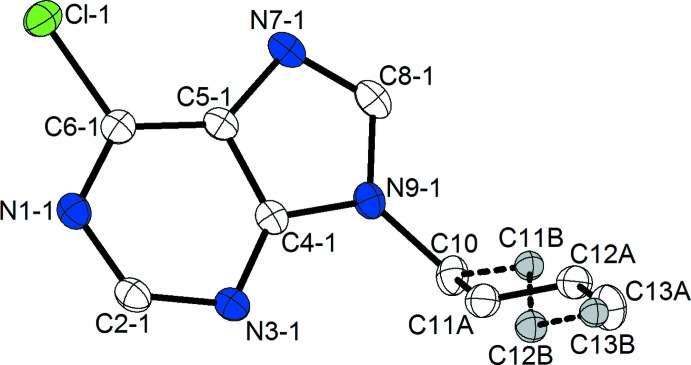
[One molecular unit of the title compound (residue 1, both A and B orientation) with atom labels and 50% probability displacement ellipsoids. Hydrogen atoms are omitted for clarity.]

**Fig. 2.**
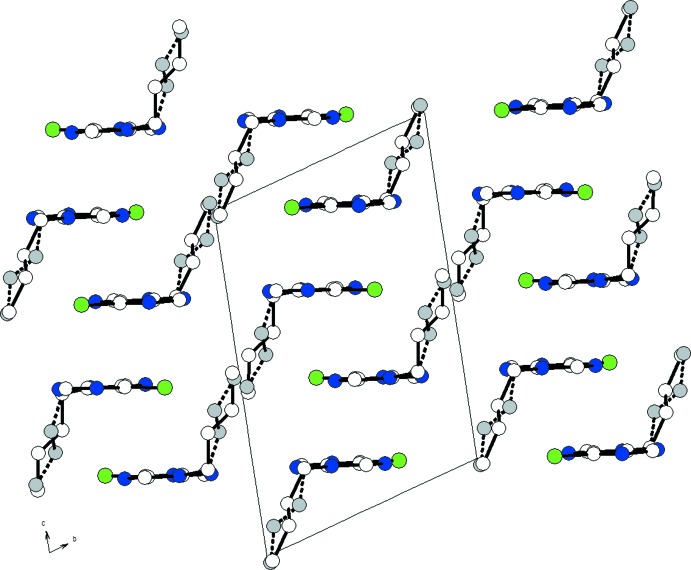
[Packing diagram of the title compound viewed along the a axis.]

**Fig. 3.**
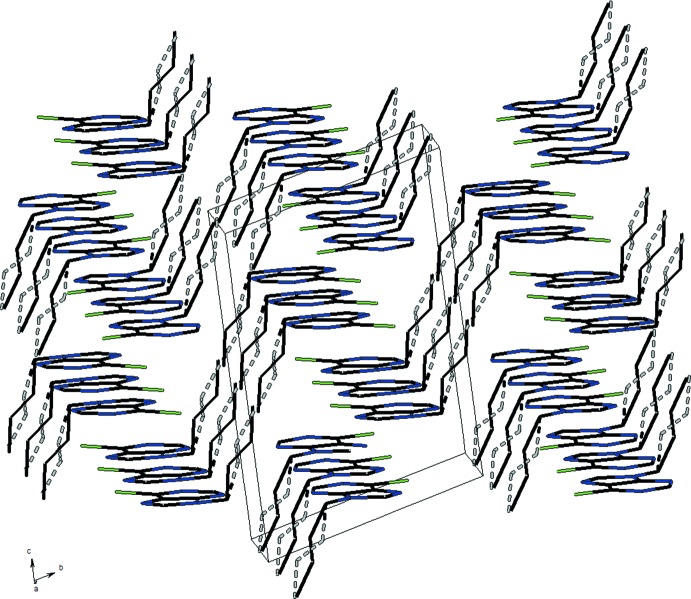
[Graphical projection of the packing of (E)-9-(but-2-en-1-yl)-6-chloro-9H-purine, C9H9ClN4.]

## supplementary crystallographic information

### Comment

The title compound is an *N*-allyl substituted purine intended as a starting material for investigation of the double bond rearragement in *N*-allylic systems. (*E*)-9-(but-2-en-1-yl)-6-chloro-9*H*-purine was recrystallized from a mixture of *E*/*Z*-isomers in order to obtain the pure *E*-isomer for the following rearrangement study. The structure and purity of this compound was originally determined by NMR. Further structure elucidation by SXRD supports the conclusions in our previous report (compound 14*a*, Kania *et al.*, 2013).

The structure of the title compound, C_9_H_9_ClN_4_, has a triclinic P -1 symmetry. The asymmetric unit consists of two molecules of the title compound, with the full content of the unit cell generated by symmetry operations. The molecule has a planar bicyclic motif with an *N*-allylic chain oriented out of the plane described by the bicyclic main body. During the initial refinement of the structure it became apparent that the *N*-allylic chain is slightly disordered, with two possible spacial orientations. By using the PART instruction in *SHELXL*, the two possible orientations of the chain (A and B) were refined individually for the two molecules that form the asymmetric unit (residue 1 and 2). Orientation A is clearly preferred in the solid state, with the minor orientation B present in about 13% and 6.6% abundance for residue 1 and 2 respectively. The limited occupancy of the minor components prompted the use of restraints to achieve satisfactory refinement of the structure. We employed the SADI instruction in *SHELXL* and restrained the C10, C11B—C13B and C20, C21B—C23B bond distances in the minor components to be the same as the corresponding distances in the major components (C10, C11A—C13A and C20, C21A—C23A). This ensured realistic bond distances in the chains. Furthermore, C11B—C13B and C21B—C23B were refined isotropically since anisotropic refinement did not give realistic thermal parameters due to the low occupancy of the sites.

### Experimental

The title compound was synthesized by the method described in Kania *et al.* (2013) (compound 14*a*).

### Refinement

H-atoms were positioned geometrically at distances of 0.95 Å (CH), 0.99 Å (CH_2_) and 0.98 Å (CH_3_), and refined using a riding model with *U*_iso_ (H)=1.2 *U*_eq_ (CH and CH_2_) and *U*_iso_ (H)=1.5 *U*_eq_ (CH_3_)

### Crystal data

**Table:**

C_9_H_9_ClN_4_	*Z* = 4
*M**_r_* = 208.65	*F*(000) = 432
Triclinic, *P*1	*D*_x_ = 1.462 Mg m^−^^3^
Hall symbol: -P 1	Melting point: 339 K
*a* = 8.1818 (11) Å	Mo *K*α radiation, λ = 0.71073 Å
*b* = 9.7103 (13) Å	Cell parameters from 8169 reflections
*c* = 13.9435 (19) Å	θ = 2.5–28.8°
α = 69.642 (1)°	µ = 0.37 mm^−^^1^
β = 75.448 (1)°	*T* = 100 K
γ = 67.032 (1)°	Rod, colourless
*V* = 947.6 (2) Å^3^	1.13 × 0.35 × 0.22 mm

### Data collection

**Table:**

Bruker APEXII CCD diffractometer	4428 independent reflections
Radiation source: fine-focus sealed tube	4103 reflections with *I* > 2σ(*I*)
Graphite monochromator	*R*_int_ = 0.017
φ and ω scans	θ_max_ = 28.8°, θ_min_ = 1.6°
Absorption correction: numerical (*SADABS*; Bruker, 2005)	*h* = −11→10
*T*_min_ = 0.683, *T*_max_ = 0.924	*k* = −12→12
10801 measured reflections	*l* = −18→18

### Refinement

**Table:**

Refinement on *F*^2^	Primary atom site location: structure-invariant direct methods
Least-squares matrix: full	Secondary atom site location: difference Fourier map
*R*[*F*^2^ > 2σ(*F*^2^)] = 0.027	Hydrogen site location: inferred from neighbouring sites
*wR*(*F*^2^) = 0.070	H-atom parameters constrained
*S* = 1.04	*w* = 1/[σ^2^(*F*_o_^2^) + (0.0292*P*)^2^ + 0.3823*P*] where *P* = (*F*_o_^2^ + 2*F*_c_^2^)/3
4428 reflections	(Δ/σ)_max_ = 0.001
283 parameters	Δρ_max_ = 0.34 e Å^−^^3^
4 restraints	Δρ_min_ = −0.30 e Å^−^^3^

### Special details

**Table:**

Experimental. Due to the weak scattering nature of the compound, a large crystal (1.13x0.35x0.22 mm) was used to get satisfactory data for refinement.
Geometry. All e.s.d.'s (except the e.s.d. in the dihedral angle between two l.s. planes) are estimated using the full covariance matrix. The cell e.s.d.'s are taken into account individually in the estimation of e.s.d.'s in distances, angles and torsion angles; correlations between e.s.d.'s in cell parameters are only used when they are defined by crystal symmetry. An approximate (isotropic) treatment of cell e.s.d.'s is used for estimating e.s.d.'s involving l.s. planes.
Refinement. Refinement of *F*^2^ against ALL reflections. The weighted *R*-factor *wR* and goodness of fit *S* are based on *F*^2^, conventional *R*-factors *R* are based on *F*, with *F* set to zero for negative *F*^2^. The threshold expression of *F*^2^ > σ(*F*^2^) is used only for calculating *R*-factors(gt) *etc*. and is not relevant to the choice of reflections for refinement. *R*-factors based on *F*^2^ are statistically about twice as large as those based on *F*, and *R*- factors based on ALL data will be even larger.

### Fractional atomic coordinates and isotropic or equivalent isotropic displacement parameters (Å^2^)

**Table:**

	*x*	*y*	*z*	*U*_iso_*/*U*_eq_	Occ. (<1)
Cl-1	0.80714 (4)	0.34683 (3)	0.90852 (2)	0.02519 (8)	
Cl-2	0.56350 (4)	0.66115 (3)	0.58169 (2)	0.02513 (8)	
N1-1	0.68069 (12)	0.64840 (11)	0.83023 (7)	0.02118 (19)	
N3-1	0.37832 (12)	0.80607 (11)	0.79779 (7)	0.01992 (18)	
N9-1	0.18663 (12)	0.65085 (11)	0.83852 (7)	0.01988 (18)	
N7-1	0.38398 (13)	0.41103 (11)	0.89887 (7)	0.02212 (19)	
N9-2	0.18425 (12)	0.33227 (11)	0.67452 (7)	0.01990 (18)	
N3-2	0.49116 (12)	0.19050 (10)	0.70937 (7)	0.02115 (19)	
N1-2	0.66555 (13)	0.36050 (11)	0.66514 (7)	0.02249 (19)	
N7-2	0.19524 (13)	0.57777 (11)	0.61328 (7)	0.0240 (2)	
C2-1	0.54905 (15)	0.78443 (13)	0.79925 (8)	0.0217 (2)	
H2-1	0.5818	0.8756	0.7757	0.026*	
C4-1	0.34503 (14)	0.67189 (12)	0.83172 (7)	0.0180 (2)	
C10	0.01759 (15)	0.77488 (14)	0.81086 (8)	0.0229 (2)	
H10A	−0.0707	0.7281	0.8113	0.028*	
H10B	0.0382	0.8425	0.7403	0.028*	
C11A	−0.05515 (18)	0.87065 (15)	0.88579 (10)	0.0221 (3)	0.870 (4)
H11A	0.0139	0.9269	0.8901	0.027*	0.870 (4)
C12A	−0.21002 (18)	0.88140 (15)	0.94633 (10)	0.0218 (3)	0.870 (4)
H12A	−0.2772	0.8236	0.9419	0.026*	0.870 (4)
C13A	−0.2880 (4)	0.9771 (4)	1.0211 (2)	0.0282 (7)	0.870 (4)
H13A	−0.3312	0.9147	1.0874	0.042*	0.870 (4)
H13B	−0.3880	1.0691	0.9940	0.042*	0.870 (4)
H13C	−0.1958	1.0101	1.0308	0.042*	0.870 (4)
C11B	−0.1202 (11)	0.8205 (9)	0.9029 (6)	0.015 (2)*	0.130 (4)
H11B	−0.1846	0.7520	0.9435	0.018*	0.130 (4)
C12B	−0.1540 (11)	0.9477 (9)	0.9284 (6)	0.018 (2)*	0.130 (4)
H12B	−0.1012	1.0218	0.8819	0.022*	0.130 (4)
C13B	−0.267 (3)	0.987 (3)	1.0233 (14)	0.015 (4)*	0.130 (4)
H13D	−0.3374	0.9172	1.0560	0.022*	0.130 (4)
H13E	−0.3476	1.0951	1.0050	0.022*	0.130 (4)
H13F	−0.1897	0.9748	1.0714	0.022*	0.130 (4)
C6-1	0.63857 (14)	0.51980 (12)	0.86514 (8)	0.0193 (2)	
C8-1	0.21905 (16)	0.49369 (13)	0.87965 (8)	0.0230 (2)	
H8-1	0.1289	0.4480	0.8932	0.028*	
C5-1	0.46671 (14)	0.52249 (12)	0.86842 (8)	0.0183 (2)	
C20	0.11732 (15)	0.20286 (13)	0.69402 (8)	0.0215 (2)	
H20A	−0.0036	0.2258	0.7343	0.026*	
H20B	0.1973	0.1061	0.7357	0.026*	
C21A	0.10844 (17)	0.17794 (14)	0.59490 (9)	0.0218 (3)	0.934 (3)
H21A	0.0198	0.2537	0.5533	0.026*	0.934 (3)
C22A	0.21845 (17)	0.05548 (15)	0.56322 (9)	0.0238 (3)	0.934 (3)
H22A	0.3056	−0.0205	0.6058	0.029*	0.934 (3)
C23A	0.2142 (4)	0.0289 (3)	0.4634 (2)	0.0325 (6)	0.934 (3)
H23A	0.1207	0.1167	0.4264	0.049*	0.934 (3)
H23B	0.1884	−0.0676	0.4786	0.049*	0.934 (3)
H23C	0.3307	0.0203	0.4204	0.049*	0.934 (3)
C21B	0.209 (2)	0.1272 (18)	0.6064 (11)	0.015 (4)*	0.066 (3)
H21B	0.3352	0.0771	0.5981	0.018*	0.066 (3)
C22B	0.112 (2)	0.1321 (17)	0.5430 (11)	0.015 (4)*	0.066 (3)
H22B	−0.0106	0.1928	0.5380	0.018*	0.066 (3)
C23B	0.229 (5)	0.026 (4)	0.481 (3)	0.021 (8)*	0.066 (3)
H23D	0.1854	0.0602	0.4137	0.031*	0.066 (3)
H23E	0.2261	−0.0801	0.5169	0.031*	0.066 (3)
H23F	0.3515	0.0260	0.4695	0.031*	0.066 (3)
C4-2	0.35605 (14)	0.31944 (12)	0.67688 (8)	0.0183 (2)	
C2-2	0.63962 (15)	0.22142 (13)	0.70213 (9)	0.0240 (2)	
H2-2	0.7401	0.1342	0.7261	0.029*	
C6-2	0.52708 (15)	0.48383 (12)	0.63305 (8)	0.0194 (2)	
C5-2	0.36118 (14)	0.47249 (12)	0.63838 (8)	0.0188 (2)	
C8-2	0.09700 (15)	0.48857 (13)	0.63580 (9)	0.0244 (2)	
H8-2	−0.0256	0.5292	0.6260	0.029*	

### Atomic displacement parameters (Å^2^)

**Table:**

	*U*^11^	*U*^22^	*U*^33^	*U*^12^	*U*^13^	*U*^23^
Cl-1	0.02417 (14)	0.02408 (14)	0.02272 (13)	−0.00338 (10)	−0.00490 (10)	−0.00524 (10)
Cl-2	0.03597 (16)	0.02178 (14)	0.02075 (13)	−0.01493 (11)	−0.00189 (10)	−0.00491 (10)
N1-1	0.0223 (5)	0.0252 (5)	0.0193 (4)	−0.0112 (4)	−0.0031 (3)	−0.0061 (4)
N3-1	0.0234 (5)	0.0209 (4)	0.0174 (4)	−0.0099 (4)	−0.0032 (3)	−0.0045 (3)
N9-1	0.0194 (4)	0.0244 (5)	0.0193 (4)	−0.0103 (4)	−0.0013 (3)	−0.0077 (3)
N7-1	0.0272 (5)	0.0224 (5)	0.0196 (4)	−0.0133 (4)	0.0011 (4)	−0.0066 (4)
N9-2	0.0189 (4)	0.0207 (4)	0.0199 (4)	−0.0057 (3)	−0.0028 (3)	−0.0063 (3)
N3-2	0.0211 (4)	0.0181 (4)	0.0223 (4)	−0.0046 (4)	−0.0039 (3)	−0.0048 (3)
N1-2	0.0213 (4)	0.0238 (5)	0.0229 (5)	−0.0082 (4)	−0.0017 (4)	−0.0074 (4)
N7-2	0.0250 (5)	0.0199 (5)	0.0249 (5)	−0.0029 (4)	−0.0077 (4)	−0.0059 (4)
C2-1	0.0259 (5)	0.0226 (5)	0.0202 (5)	−0.0128 (4)	−0.0037 (4)	−0.0045 (4)
C4-1	0.0206 (5)	0.0229 (5)	0.0134 (4)	−0.0098 (4)	−0.0012 (4)	−0.0063 (4)
C10	0.0191 (5)	0.0302 (6)	0.0210 (5)	−0.0089 (4)	−0.0039 (4)	−0.0075 (4)
C11A	0.0226 (7)	0.0210 (6)	0.0243 (6)	−0.0085 (5)	−0.0078 (5)	−0.0036 (5)
C12A	0.0236 (7)	0.0205 (6)	0.0226 (6)	−0.0086 (5)	−0.0066 (5)	−0.0037 (5)
C13A	0.0264 (11)	0.0339 (12)	0.0311 (11)	−0.0128 (9)	−0.0020 (7)	−0.0152 (7)
C6-1	0.0216 (5)	0.0220 (5)	0.0143 (4)	−0.0068 (4)	−0.0027 (4)	−0.0056 (4)
C8-1	0.0270 (6)	0.0263 (6)	0.0213 (5)	−0.0158 (5)	0.0021 (4)	−0.0091 (4)
C5-1	0.0229 (5)	0.0198 (5)	0.0138 (4)	−0.0094 (4)	−0.0004 (4)	−0.0056 (4)
C20	0.0218 (5)	0.0242 (5)	0.0203 (5)	−0.0106 (4)	−0.0005 (4)	−0.0066 (4)
C21A	0.0210 (6)	0.0247 (6)	0.0205 (6)	−0.0097 (5)	−0.0039 (4)	−0.0039 (4)
C22A	0.0253 (6)	0.0246 (6)	0.0212 (6)	−0.0098 (5)	−0.0033 (5)	−0.0043 (5)
C23A	0.0420 (11)	0.0391 (10)	0.0225 (10)	−0.0165 (7)	−0.0021 (8)	−0.0141 (8)
C4-2	0.0199 (5)	0.0204 (5)	0.0145 (4)	−0.0065 (4)	−0.0012 (4)	−0.0059 (4)
C2-2	0.0215 (5)	0.0204 (5)	0.0277 (6)	−0.0046 (4)	−0.0050 (4)	−0.0056 (4)
C6-2	0.0265 (5)	0.0194 (5)	0.0140 (4)	−0.0097 (4)	−0.0004 (4)	−0.0059 (4)
C5-2	0.0227 (5)	0.0177 (5)	0.0149 (4)	−0.0048 (4)	−0.0026 (4)	−0.0054 (4)
C8-2	0.0218 (5)	0.0233 (5)	0.0262 (5)	−0.0021 (4)	−0.0072 (4)	−0.0079 (4)

### Geometric parameters (Å, º)

**Table:**

Cl-1—C6-1	1.7302 (11)	C13A—H13C	0.9800
Cl-2—C6-2	1.7325 (11)	C11B—C12B	1.311 (11)
N1-1—C6-1	1.3205 (14)	C11B—H11B	0.9500
N1-1—C2-1	1.3483 (15)	C12B—C13B	1.487 (15)
N3-1—C2-1	1.3334 (14)	C12B—H12B	0.9500
N3-1—C4-1	1.3338 (13)	C13B—H13D	0.9800
N9-1—C4-1	1.3644 (13)	C13B—H13E	0.9800
N9-1—C8-1	1.3711 (14)	C13B—H13F	0.9800
N9-1—C10	1.4687 (14)	C6-1—C5-1	1.3856 (15)
N7-1—C8-1	1.3130 (15)	C8-1—H8-1	0.9500
N7-1—C5-1	1.3847 (13)	C20—C21A	1.5069 (15)
N9-2—C4-2	1.3697 (14)	C20—C21B	1.526 (15)
N9-2—C8-2	1.3721 (14)	C20—H20A	0.9900
N9-2—C20	1.4756 (14)	C20—H20B	0.9900
N3-2—C4-2	1.3311 (14)	C21A—C22A	1.3227 (18)
N3-2—C2-2	1.3333 (15)	C21A—H21A	0.9500
N1-2—C6-2	1.3195 (15)	C22A—C23A	1.513 (3)
N1-2—C2-2	1.3486 (15)	C22A—H22A	0.9500
N7-2—C8-2	1.3123 (15)	C23A—H23A	0.9800
N7-2—C5-2	1.3848 (14)	C23A—H23B	0.9800
C2-1—H2-1	0.9500	C23A—H23C	0.9800
C4-1—C5-1	1.4035 (15)	C21B—C22B	1.308 (15)
C10—C11A	1.4975 (17)	C21B—H21B	0.9500
C10—C11B	1.547 (8)	C22B—C23B	1.475 (18)
C10—H10A	0.9900	C22B—H22B	0.9500
C10—H10B	0.9900	C23B—H23D	0.9800
C11A—C12A	1.323 (2)	C23B—H23E	0.9800
C11A—H11A	0.9500	C23B—H23F	0.9800
C12A—C13A	1.494 (3)	C4-2—C5-2	1.4075 (15)
C12A—H12A	0.9500	C2-2—H2-2	0.9500
C13A—H13A	0.9800	C6-2—C5-2	1.3857 (15)
C13A—H13B	0.9800	C8-2—H8-2	0.9500
			
C6-1—N1-1—C2-1	117.58 (9)	N1-1—C6-1—C5-1	121.81 (10)
C2-1—N3-1—C4-1	111.92 (9)	N1-1—C6-1—Cl-1	117.06 (8)
C4-1—N9-1—C8-1	105.55 (9)	C5-1—C6-1—Cl-1	121.12 (8)
C4-1—N9-1—C10	125.75 (9)	N7-1—C8-1—N9-1	114.96 (10)
C8-1—N9-1—C10	128.65 (9)	N7-1—C8-1—H8-1	122.5
C8-1—N7-1—C5-1	103.03 (9)	N9-1—C8-1—H8-1	122.5
C4-2—N9-2—C8-2	105.39 (9)	N7-1—C5-1—C6-1	135.09 (10)
C4-2—N9-2—C20	126.48 (9)	N7-1—C5-1—C4-1	110.75 (9)
C8-2—N9-2—C20	127.49 (9)	C6-1—C5-1—C4-1	114.16 (9)
C4-2—N3-2—C2-2	111.78 (9)	N9-2—C20—C21A	111.69 (9)
C6-2—N1-2—C2-2	116.99 (10)	N9-2—C20—C21B	106.9 (6)
C8-2—N7-2—C5-2	103.20 (9)	N9-2—C20—H20A	109.3
N3-1—C2-1—N1-1	127.67 (10)	C21A—C20—H20A	109.3
N3-1—C2-1—H2-1	116.2	C21B—C20—H20A	135.2
N1-1—C2-1—H2-1	116.2	N9-2—C20—H20B	109.3
N3-1—C4-1—N9-1	127.49 (10)	C21A—C20—H20B	109.3
N3-1—C4-1—C5-1	126.81 (10)	C21B—C20—H20B	83.6
N9-1—C4-1—C5-1	105.70 (9)	H20A—C20—H20B	107.9
N9-1—C10—C11A	110.39 (9)	C22A—C21A—C20	123.02 (11)
N9-1—C10—C11B	115.3 (3)	C22A—C21A—H21A	118.5
N9-1—C10—H10A	109.6	C20—C21A—H21A	118.5
C11A—C10—H10A	109.6	C21A—C22A—C23A	124.07 (14)
C11B—C10—H10A	80.2	C21A—C22A—H22A	118.0
N9-1—C10—H10B	109.6	C23A—C22A—H22A	118.0
C11A—C10—H10B	109.6	C22B—C21B—C20	119.2 (14)
C11B—C10—H10B	128.2	C22B—C21B—H21B	120.4
H10A—C10—H10B	108.1	C20—C21B—H21B	120.4
C12A—C11A—C10	123.78 (12)	C21B—C22B—C23B	107.1 (19)
C12A—C11A—H11A	118.1	C21B—C22B—H22B	126.5
C10—C11A—H11A	118.1	C23B—C22B—H22B	126.5
C11A—C12A—C13A	125.33 (17)	N3-2—C4-2—N9-2	127.88 (10)
C11A—C12A—H12A	117.3	N3-2—C4-2—C5-2	126.46 (10)
C13A—C12A—H12A	117.3	N9-2—C4-2—C5-2	105.66 (9)
C12B—C11B—C10	124.2 (8)	N3-2—C2-2—N1-2	128.37 (10)
C12B—C11B—H11B	117.9	N3-2—C2-2—H2-2	115.8
C10—C11B—H11B	117.9	N1-2—C2-2—H2-2	115.8
C11B—C12B—C13B	126.3 (11)	N1-2—C6-2—C5-2	121.91 (10)
C11B—C12B—H12B	116.9	N1-2—C6-2—Cl-2	116.70 (8)
C13B—C12B—H12B	116.9	C5-2—C6-2—Cl-2	121.39 (8)
C12B—C13B—H13D	109.5	N7-2—C5-2—C6-2	134.92 (10)
C12B—C13B—H13E	109.5	N7-2—C5-2—C4-2	110.64 (9)
H13D—C13B—H13E	109.5	C6-2—C5-2—C4-2	114.43 (9)
C12B—C13B—H13F	109.5	N7-2—C8-2—N9-2	115.10 (10)
H13D—C13B—H13F	109.5	N7-2—C8-2—H8-2	122.4
H13E—C13B—H13F	109.5	N9-2—C8-2—H8-2	122.4
